# Revisiting the Policy Implications of Medical Tourism in the Post-COVID-19 Pandemic From a Malaysian Perspective: A Qualitative Study

**DOI:** 10.7759/cureus.64308

**Published:** 2024-07-11

**Authors:** Mohd Fauzy Samsudin, Yin Cheng Lim, Thinni Nurul Rochmah, Maznah Dahlui

**Affiliations:** 1 Department of Social and Preventive Medicine, Faculty of Medicine, University of Malaya, Kuala Lumpur, MYS; 2 Department of Health Administration and Policy, University of Airlangga, Surabaya, IDN; 3 Department of Research Development and Innovation, University of Malaya Medical Centre, Kuala Lumpur, MYS

**Keywords:** qualitative study, trade in health services, health policy, healthcare system, medical tourism

## Abstract

Background

Malaysia has been one of the most prominent destination countries for medical tourism. The industry received significant government support to create a conducive environment for its growth, such as the provision of an investment tax allowance for the facilities participating in medical tourism and the establishment of the Malaysia Healthcare Travel Council (MHTC) to coordinate collaboration between various industry stakeholders to promote medical tourism activities at the international level and facilitate inbound medical tourists. The establishment of the MHTC facilitates collaboration between various industry stakeholders. In addition to facilitating medical tourism activities, MHTC is also involved in analysing relevant data, including but not limited to the socio-demography of medical tourists, the trend of their healthcare service utilisation, revenue generated, and market intelligence to promote the industry's growth. The council serves as a medium to facilitate the collaboration of stakeholders such as the hospitals participating in medical tourism, the Association of Private Hospitals Malaysia, the Malaysian Society for Quality in Health, and various government agencies, including the Ministry of Health and the Department of Immigration, Malaysia. We explore the policy-related implications of medical tourism and its relationship with the Malaysian national healthcare system.

Methodology

We revisited Pocock and Phua’s conceptual framework of policy implications for medical tourism to explore its relevance after more than a decade of intensive government support and the aftermath of the COVID-19 pandemic. We employed a qualitative case study design using semi-structured, in-depth interviews with stakeholders from the Ministry of Health Malaysia, the private health sector, professional bodies, academics, and health-related civil society organisations.

Results

Our study found that many issues identified in the original framework remain relevant after over a decade. However, we also identified additional implications, such as the role of insurance portability in encouraging the growth of international hospital accreditation and the issue of equitable access to healthcare within the context of the current Malaysian healthcare system in the aftermath of COVID-19.

Conclusion

Due to its vulnerability, there is a need to develop a strategic collaboration that includes incorporating medical tourism activities into a broader framework, such as promoting aged care within the retirement destinations package for expatriates, which could ensure its sustainability instead of relying solely on medical tourism activities. In the meantime, policy implications arising from the industry remain relevant and should be addressed through a comprehensive structural reform of the national healthcare system involving stakeholders from the public and private health sectors.

## Introduction

Medical tourism is a global phenomenon that generally refers to the movement of patients across an international border to seek medical care. However, there is no consistent definition of medical tourism [1], since there are no standardised concepts and definitions that encompass the industry [2]. Although there is no general consensus on the definition, medical tourism is widely accepted as an act when consumers elect to travel across international borders with the intention of receiving some form of medical treatment, including but not limited to dental care, elective surgery, and fertility treatment. It is also defined as ‘the organised travel outside one’s natural healthcare jurisdiction for the enhancement or restoration of the individual’s health through medical intervention’ [3], associated with higher quality or more accessibility than one’s country of origin [4]. On a different note, medical tourism is also considered a subcategory of medical travel, in which ‘patient-tourists’ travel for elective care of their own volition [5]. Some authors included a link between the purpose of seeking care and ‘direct or indirect engagement of leisure business’ [6] ‘to ease foreign patients into a new cultural environment and to occupy them during the pre- and post-operative periods’ [7], where ‘accompanying relatives or friends may be more engaged in tourism activities’ [8]. It is a relatively new industry, and the emerging global economic sector is rapidly gaining momentum due to its economic potential, which provides lucrative business opportunities to key players in the industry. The rapid growth of this industry can be seen over the last few decades with the development of better transportation and increased mobility in the era of globalisation.

Medical tourism in Malaysia was first identified as a growing sector in 1997, after the Asian financial crisis, when there was a significant surge in the number of Indonesian patients seeking treatment from Malaysian private hospitals for affordable and quality healthcare [9]. During the financial crisis, the Malaysian private healthcare sector was adversely affected due to decreased patient load as healthcare consumers reverted to utilising public health facilities, which saw an 18 per cent increase in patients [10]. The private hospitals responded by seeking measures to expand the market by promoting their underutilised services abroad to attract consumers from other countries. Thus, the main motivation for these private hospitals to venture into this industry was to fill in the excess capacity within their facilities. In Malaysia, private hospitals are allowed to promote their services based on the Advertising Guidelines for Healthcare Facilities and Services (Private Hospitals, Clinics, and Medical Laboratories) [11]. Health services advertisement is governed by the Medicine Advertisements Board (MAB) Regulations 1976. To advertise their services for treatment, prevention, or diagnosis of diseases, they must apply for MAB approval. Failure to comply is an offence under Section 4A of the Medicines (Advertisement and Sale) Act 1956.

Following the economic crisis in 1997, the government saw the potential of medical tourism as an economic sector. It began capitalising on this industry to integrate Malaysia further into the global healthcare marketplace. In addition to health service advertisements by private hospitals, the inter-ministerial National Committee for the Promotion of Health Tourism in Malaysia was also established in January 1998 to promote medical and health tourism. This committee had five subcommittees to identify suitable departure countries for promoting medical tourism, planning for tax incentives and fee packaging, and developing guidelines for accreditation and advertisement [12]. In 2009, the Malaysia Healthcare Travel Council (MHTC) was established to promote Malaysia as a destination for high-quality medical tourism at an affordable price and to facilitate collaboration between government and industry players among private hospitals [13].

However, there is a concern about medical tourism's impact on the provision of and access to quality healthcare services among the local populations. To date, this industry only involves providers from the private health sector. Even though there are no substantial differences in technical aspects, the public health sector tends to lag behind the private sector in a few areas, especially in terms of long waiting times and the physical conditions of the health facilities [14]. It is of concern that medical tourists would siphon healthcare resources and gain access to better-quality services compared to locals, especially those from a lower socio-economic status [15]. This situation can worsen if proper healthcare reforms are not implemented to bridge the gap and tackle the inequity between the public and private sectors in Malaysia’s dichotomous two-tier healthcare system.

Overview of the Malaysian healthcare system

The Malaysian healthcare system exhibits a dichotomous two-tier model comprising the public sector, which is government-funded by general taxation, and the private sector. Public healthcare providers in Malaysia charge a nominal fee or provide exemptions for patients. This approach aims to serve the poor and rural populations, ensuring equitable access for those in need. Conversely, private sector providers rely mainly on out-of-pocket (OOP) payments and private insurance and are typically found in urban areas [16]. However, the government's overemphasis on the physical coverage of services contributed to higher costs and compromised allocative and technical efficiency. These inefficiencies in the system have contributed to greater inequity in other forms, despite having extensive physical coverage [10].

The private sector thrived over the last three decades following the Malaysian government's introduction of privatisation and corporatisation policies in the 1980s [12]. The change in government policy, rapid urbanisation, and the emergence of the middle classes led to the growth of corporate, investor-owned private hospitals. In response to the rapid growth of private hospitals, the Private Healthcare Facilities and Services Act (PHFSA 1998) was introduced to regulate the private health sector. The privatisation of healthcare during this period served as the foundation upon which the medical tourism industry in Malaysia began to grow.

This two-tiered system would become more pronounced as the proportion of OOP payments increases, which could create polarisation where the poor utilise government services whilst those with higher incomes opt for private health services. Eventually, this could lead to inequitable access to healthcare as the public hospitals are overcrowded by the majority of those from lower socio-economic status and have longer waiting times, whereas those with higher purchasing power choose private hospitals [17,18]. This would, therefore, undermine the principle, which serves as the basis of the Malaysian healthcare system, that access to healthcare should not be based on the ability to pay [16].

Medical tourism industry within the Malaysian context post-COVID-19 pandemic

Following a decade of collaborative efforts to promote the industry, medical tourism in Malaysia has grown extensively, with a total of 1.22 million inbound healthcare travellers reported in 2019, generating double-digit growth in revenue between 2015 (Malaysian Ringgit, MYR 954 million) and 2019 (MYR 1.7 billion) and a compounded annual growth rate of 16.3% between 2015 and 2019. Top medical procedures sought after in Malaysia by medical tourists include but are not limited to obstetrics and gynaecology, cardiology, ophthalmology, aesthetics, oncology, orthopaedics, and fertility procedures [19].

However, the outbreak of the COVID-19 pandemic has had an adverse impact on medical tourism. Travel restrictions imposed following the pandemic in 2020 have adversely affected various industries dependent on cross-border trade and services, including medical tourism. Despite the travel restrictions, the demands from medical tourists were still available. As countries began to ease up the lockdowns cautiously and mutually create ‘travel bubbles’ that allow limited travel between selected countries, the industry started to embark on the gradual path towards recovery. Although the gradual reopening of borders for these inbound medical tourists has already taken place as early as June 2020, it is anticipated that the industry may take years to recover. Medical tourists dropped to 689,000 in 2020 from 1.22 million in 2019. These included medical tourists who travelled before the travel restriction and stayed in Malaysia under the Malaysia My Second Home programme. These numbers also included those who needed inaccessible or relatively more expensive critical care in their country of origin through the humanitarian ‘travel bubbles’ on a case-to-case basis. Meanwhile, the revenue generated from the industry dropped 54 per cent to MYR 782 million in 2020 compared to 2019 [20]. The indirect impact of the economic downturn following the pandemic may also result in a knock-on effect on the medical tourism sector, potentially devastating the industry.

Study rationale

In response to the escalating healthcare costs and increasing burden of non-communicable diseases in this country, Malaysia has long planned healthcare reforms to ensure sustainable and equitable healthcare service delivery. Over the recent decades, Malaysia has quickly become an ageing nation, and this demographic transition presents a huge challenge to the healthcare system if no appropriate planning is done for its reforms. Meanwhile, the public health sector bears a significant burden on the provision of healthcare for the elderly population in outpatient and inpatient healthcare services [21]. Furthermore, ageing is associated with higher morbidity, higher utilisation of healthcare services, and increasing demand for specialised care. Hence, the ageing population may consequently lead to increasing demand for social support and healthcare services, which will, in turn, result in the consumption of a large portion of funds allocated for healthcare expenditure [22]. This trend poses challenges to our current healthcare system since most of the population with lower economic status seeks treatment from government healthcare facilities as they cannot afford to cover the cost of treatment in private healthcare services.

These factors, in combination with economic uncertainties in recent years, may contribute to inequities in access to healthcare delivery services in our dichotomous public-private healthcare system in terms of the unequal distribution of patient burden between both sectors. Thus, it is becoming more challenging for the Malaysian government to sustain the current health financing mechanism, which is largely based on funding from general taxation. This may push for a call for the privatisation of public health facilities. This will inarguably shift the direction of healthcare reforms in Malaysia from a ‘welfarist’ policy towards fostering privatised healthcare providers [23]. This study aims to explore and understand medical tourism's existing and potential implications for the healthcare system from the stakeholders’ point of view. It also helps to understand and explain how institutional characteristics shape the demand for and implications of medical tourism for the Malaysian national healthcare system.

## Materials and methods

We conducted a series of semi-structured, in-depth interviews within a qualitative case study methodological framework from 2021 to 2022 in Kuala Lumpur, Putrajaya, Selangor, and Perak, Malaysia, to better understand the policy implications of medical tourism within the context of the Malaysian healthcare system. The Medical Research and Ethics Committee of the Ministry of Health Malaysia has provided ethical approval for this study (approval number: NMRR-19-2888-45815).

Study population

Based on the literature review, we first developed a list of established and potential stakeholders and collectively finalised it. We carefully considered the informants' diversity to capture a wide breadth of perspectives, thus allowing us to explore differences and similarities across these various stakeholders. Key informants were selected from the representatives of stakeholders from relevant government agencies (Policy and International Relations Division and National Health Financing Unit, Planning Division, Ministry of Health; and MHTC), industry players (Chief Executive Officers and Medical Director of private hospitals in Malaysia), medical professional bodies (Association of Private Hospitals Malaysia and Malaysia Medical Association), academics (professor and senior lecturers from business and administration faculty at local public universities), health-related civil society organisations (medical professionals, and a lawyer from a local medical section of non-governmental organisations and research officers from health-related non-profit institutes), and medical tourists. The opinions provided by the informants were their personal views and do not necessarily represent the official stance of their organisation unless stated otherwise.

Selection criteria

The potential informants representing any organisations were selected based on at least one of these criteria: (1) that their organisation has direct involvement in the planning, coordination, implementation, promotion, and facilitation of the medical tourism industry in Malaysia; (2) involved in monitoring, evaluation, or medical tourism and healthcare system research; or (3) actively participating in the advocacy of health-related issues. Additionally, consumers with a history of utilising medical tourism services at least once were included as potential informants. Subsequently, we employed a purposive, snowball sampling method to recruit the key informants for the interviews based on the ongoing referrals from the informants until data saturation was reached. Saturation is when the data collected no longer brings additional insight to answer the research questions, usually within the first twelve interviews [24].

Method and instrument of data collection

The first author conducted the interview sessions using a semi-structured interview guide, which three independent researchers reviewed for the suitability of the questions and appropriate use of language. The interview guide underwent pilot tests with two informants for their feedback on the content, its answerability, and language clarity.

The interview guide consists of four parts. The first part focused on the informant’s general opinion on the medical tourism industry in Malaysia. In the second part, we explored the informant’s views on the multi-faceted relationship between medical tourism and the national healthcare system. This part includes questions on their opinions regarding the current strengths of the industry, challenges within the healthcare system, and the existing and anticipated implications of the industry.

The second part was developed based on the existing policy implications framework of medical tourism by Pocock and Phua [25], which covers five domains: governance, healthcare delivery, healthcare financing, human resources in the health sector, and regulation of quality control and new actors. The framework was based on a retrospective literature review of the medical tourism industry in Malaysia, Singapore, and Thailand - three major destination countries for medical tourism in the Southeast Asia region. It aims to explore and organise the health policy implications of medical tourism for destination countries and to provide policy recommendations to optimise the benefits of medical tourism for both foreign and local consumers and mitigate the risks. This framework serves as a basis for future studies weighing the benefits and disadvantages of medical tourism for health systems.

The current study revisited this framework due to its comprehensive exploration of healthcare systems from a systemic perspective in an organised manner. The domain of 'governance' refers to the policy implications resulting from the government's legislation and planning on medical tourism. This involves reconciling the aims of economic growth through trade liberalisation and less government intervention with the health sector objectives for equitable health service provision, which, on the other hand, require stronger government intervention. Meanwhile, the domain of 'healthcare delivery' refers to the implications of policies that cater to medical tourism towards healthcare system provision and infrastructure. The domain of 'healthcare financing' focused on the implications of the policy that promotes medical tourism on the trend of the mode of payment and funding of the healthcare system. The domain of 'human resources in the health sector' covers the implications of policies related to medical tourism for the training, supply, and distribution of healthcare personnel. Lastly, the domain of 'regulation of quality control and new actors' focused on the formulation and enforcement of protocols and regulations to promote the growth of medical tourism whilst ensuring the provision of quality health provision.

Meanwhile, the third part of the guide covered the informant’s opinions on the impact of COVID-19 on the dynamics of the relationship between medical tourism and the healthcare system. In the final part, we asked the informant to provide concluding remarks on how they or their organisation could contribute to a conducive environment for medical tourism to grow within a better healthcare system.

A total of 34 potential informants were approached by phone and email to participate in the interview at a time and location based on their convenience. The first author provided a detailed explanation of the purpose and conduct of the study to the informants. They were also briefed on the study's benefits and risks and provided ample time to ask questions for clarification. A copy of the participant information sheet containing the details of the study was also provided to the informants with a consent form.

In total, 22 informants responded and consented to participate in this study. Eleven of these informants agreed to face-to-face interviews, whilst the remaining 10 opted for online video conferences. However, an informant withdrew from the interview before its completion. All interviews were audio-recorded. Follow-up questions, the subsequent validation process, and ensuring rigour were conducted through online video conferencing, phone calls, or emails. Table 1 provides an overview of the informants' backgrounds and their respective professional domains.

**Table 1:** Background of the informants

Category	Number of participants	Gender		Age			Education level
		Male	Female	20-39 years old	40-59 years old	60 and above	Tertiary education
Government and public health sector	4	2	2	0	4	0	4
Academia	4	2	2	1	3	0	4
Professional organisation	2	2	0	0	1	1	2
Private health sector	3	2	1	0	2	1	3
Civil society	6	3	3	2	1	3	6
Consumer	2	0	2	0	0	2	2

Data analysis

The first author transcribed all recordings verbatim in the original language to maintain the content's closest meaning. The first author removed personal identifiers from the transcripts and replaced them with unique identification numbers. Another author subsequently validated these transcripts.

The transcripts were subsequently uploaded into NVivo version 12.0 for Windows (Lumivero, Denver, CO, USA) for thematic analysis. Thematic analysis was conducted based on the scope of the topic guides. Variables were then identified, labelled, categorised, and related together through the coding process according to the outline in the topic guide. The relationships between these categories were explored and explained to understand the multifaceted implications of medical tourism for the healthcare system. Lastly, selective coding was conducted by two data coders, inclusive of the researcher, to select and identify the core category and related categories. The first author independently identified, labelled, and categorised variables through the preliminary coding. Based on the adapted categorisation scheme from Pocock and Phua’s framework, the first author organised the emergent themes into their respective domains. The relationships between these categories were explored and explained to understand the multifaceted implications of medical tourism for the healthcare system. Opinions that did not conform to the existing categories were organised separately. The synthesis of these non-conforming findings was then discussed with other authors to achieve consensus on the comprehensiveness of the categorisation made by the primary author.

Trustworthiness of the study

Several measures were taken during interviews to ensure the validity and reliability of the findings. Additional materials were also collected to facilitate a better understanding of the informant and their contextual factors. This was done by documenting the informant’s non-verbal cues, such as facial expressions and body language, throughout the interview in the field notes. The conduct of each interview session and the researcher’s general impression of the informant were noted in a journal. These served as reflexive guides for improving the subsequent interviews and facilitating the later part of data analysis and interpretation. These measures were conducted throughout the study until data saturation was reached.

The primary researcher (who is also the first author) is a medical officer employed by the Ministry of Health Malaysia. Based on his working experience at various levels in the ministry, it is apparent from the researcher’s point of view that there is still room for improvement towards achieving universal healthcare. During the brief stint in the State Health Department, the primary researcher observed collaboration between the State Health Department and private hospitals. Despite the difference in the business model, both the public and private sectors contribute towards catering to the healthcare needs of this country. However, given the dichotomous nature of the Malaysian healthcare system, there are concerns about equity in terms of access to healthcare, including for marginalised populations. Therefore, the primary researcher may be biased in exploring the policy implications of medical tourism for the healthcare system. Nonetheless, the primary researcher’s bias was reduced through the active participation of the informants during the interview sessions. Meanwhile, we acknowledged that the informants may develop a biased perception towards him, preventing them from sharing the fullness of their experience. Therefore, he has always been explicit to the informants that his role is that of a researcher who is objectively attempting to gain better insight into the medical tourism industry and its implications for the healthcare system.

Credibility

The involvement of diverse informants in this study provided a rich understanding of the implications of medical tourism for the national healthcare system. Individual views and experiences were verified against others. They were selected from several organisations to reduce the potential effect of local factors or viewpoints peculiar to one organisation.

We also conducted analyst triangulation, in which several analysts reviewed the study findings. The primary researcher conducted the qualitative analysis independently, which was reviewed by another researcher. A table of themes was created to illustrate the collation of codes, themes, and categories. Codes and themes were also jointly discussed with the research supervisor over time.

Member checking was also conducted to enhance rigour and establish the credibility of this study. We returned the transcripts of the interviews and the summary of the interpretation of the findings to the informants for their feedback. Subsequent interviews were conducted through phone conversations or video conferences to confirm, modify, and verify the transcript.

Transferability

The primary researcher has provided a detailed description of the boundaries of this study, which was conducted to explore the existing and potential implications of medical tourism for the healthcare system within the context of Malaysia as a destination country for medical tourism. The qualitative study was conducted after the emergence of the COVID-19 pandemic, which has adversely affected medical tourism activities due to transboundary travel restrictions imposed, the gradual reopening of the borders, and the prospects of the industry after the end of the pandemic. Therefore, the viewpoints of the informants would have been affected by the sudden detrimental impact of COVID-19 on the industry. This aspect was also covered in the interviews with the informants.

Confirmability and Dependability

Preliminary data analysis was conducted concurrently with data collection through note-taking during the interview sessions. Further analysis was conducted upon the completion of each interview. The analysis was to generate more questions depending on the informants' statements. Other researchers were also involved during the analysis to provide judgements about the potential for bias or distortion. This was achieved through the examination of the data collection and analysis procedures by other researchers. Audio recordings, transcripts, written field notes, and reflexive journals were provided to them for this purpose.

Building on the categorisation adapted from Pocock and Phua’s framework of policy implications resulting from medical tourism, the emerging themes were organised into the respective domains: governance, delivery, financing, regulation, and human capital. Opinions that did not conform to the existing categories were organised separately. The synthesis of these non-conforming findings was then discussed with other authors to achieve consensus on the comprehensiveness of categorisation made by the primary author.

## Results

The findings revealed that the opinions of these informants on medical tourism varied, ranging from being fully supportive to totally against the industry. Half of the informants were fully supportive of medical tourism. On the other hand, three informants were totally against this industry. The remaining seven informants were neutral in their position towards medical tourism. The proponents of medical tourism consist of representatives from government agencies, private hospitals, medical professional bodies, a researcher, and a senior lecturer. Meanwhile, those who opposed were informants from civil societies. The remaining informants had a neutral stand on medical tourism.

Regardless of their stand towards the industry, all informants agreed that the right to access an equitable healthcare system should not be set aside to expand the medical tourism industry. The industry's supporters argued that it could enhance the quality of healthcare services and serve as a driver that could improve the well-being of the locals in general through economic growth and prosperity.

Consequently, a new theme has been agreed upon as another analytical category of ‘equitable access’. Within the context of the current study, we defined the theme 'equitable access' as the ability of patients to access the full range of quality health services based on their needs, regardless of their ability to pay. The findings from this new emerging theme are outlined along with the existing five domains identified in the original framework by Pocock and Phua. Table 2 outlines the findings of the themes and sub-themes from the current study.

**Table 2:** Themes and sub-themes

Themes	Sub-themes
Governance	Health sector commitments in trade agreements, stakeholders’ relationship, conflicting ministerial roles and responsibilities
Delivery	Private sector growth and excess capacity utilisation, government incentives, public sector involvement, for-profit foreign equity participation and competition
Financing	Redistributive financing mechanism, differential pricing, universal health coverage
Regulation	Public and private regulations and quality control, international and local accreditation bodies, enhancement of standard of care
Human capital	Distribution of health professionals between public and private, choice of training for specialisation, two-tier training along the public-private divide
Equitable access	Maldistribution of services and facilities, rationing of healthcare, polarisation in the two-tier healthcare system

Governance

The informants had mixed opinions regarding Malaysia's trade agreements. Some agreed that these may benefit the industry, whilst others expressed concern that they may be detrimental to our healthcare system. Trade liberalisation within the ASEAN Economic Community (AEC) facilitates Malaysia’s medical tourism industry through the movement of patients. Such trade agreements allow the government to tap into a wider market and promote the growth of the transboundary medical tourism industry. However, there are concerns among the informants that these agreements may allow broader foreign control of the healthcare system and further reduce the policy-making space available to the government to act on behalf of the people. Therefore, trade agreements should be carefully negotiated to protect national interests, especially in the national healthcare system, as it is a matter of national security.

We are benefitting from the trade liberalisation in ASEAN, especially in the AEC and ASEAN Economic Community, where they emphasise the movement of patients and the movement of healthcare professionals, doctors, and nurses. In terms of movement of patients, there is where it facilitates the business activities in medical tourism, in terms of visa application, transportation, those have been eased and facilitated to support medical tourism. (Academic researcher)

Additionally, an informant representing one of the stakeholders from private hospitals welcomed an agreement on the movement of persons to another country to provide a service, such as in one of the modes of supplying services under Mode 4 of the General Agreement on Trade and Services. The presence of foreign health professionals may be beneficial in promoting medical tourism to clients from the same country who share the same culture and language.

In Thailand and Singapore, the fact that they are able to have foreign doctors really helps to boost medical tourism because there’s also this communication (factor) and cultural affinity that is important when it comes to providing healthcare services. Unlike hospitality, healthcare patients from overseas are very anxious, requiring detailed explanations. It is important that people who share the same culture and language can understand to communicate these. (Chief Executive Officer of a private hospital)

However, other informants raised concerns regarding the influx of foreign healthcare workers, which could inadvertently discourage locals from venturing into the healthcare profession. It might also undermine efforts to improve the working environment and remuneration for local healthcare workers. Nonetheless, foreign health professionals must fulfil stringent requirements and sit for local examinations before practising in Malaysia to protect the interests of local health professionals. There is also concern regarding national interests. These agreements may allow broader foreign control of our healthcare system and further reduce the policy-making space available to the government to act on behalf of the people.

If you give them those (as stipulated under the trade agreements), that means they will have control of your healthcare resources. Is that a desirable thing for the government to do? You let your doctor and your resources be under the care of investors from the United States of America, for example, and have more and more say in your healthcare system. I think the government should review and be careful when signing all these international agreements to protect public health. (Senior fellow in an academic institution)

Meanwhile, the industry received significant governmental support through various ministries and agencies. Informants clarified that establishing the MHTC has further strengthened collaboration among industry stakeholders. Furthermore, the Malaysian government, through the Ministry of International Trade and Industry, has established the Malaysia Productivity Corporation, which encourages collaborative efforts among the stakeholders in reducing regulatory burden and breaking bureaucratic silos.

The good thing about Malaysia is that we have Malaysia Healthcare Travel Council, which is the main and the only sole mediator between the private hospital, the government, and the medical tourists. (Medical Director of a private hospital)

One agency you must highlight is Malaysia Productivity Corporation, under the MITI (Ministry of International Trade and Industry). One of the nexuses is the private healthcare productivity nexus. They also have the tourism nexus, but under the private healthcare productivity nexus, the purpose is to increase the efficiency that leads to productivity. They look into how to reduce regulatory burden and cut the silos. (Honorary Secretary General of a professional organisation)

On a different note, several informants highlighted the conflicting role of the Ministry of Health as the funder, regulator, and operator in healthcare delivery. In a two-tier healthcare system, the government's investment in private healthcare through sovereign funds raises concerns about conflicting interests between developing the medical tourism industry and improving the public healthcare system.

You must understand the industry players; their main goal is to generate enough profits for the shareholders. So, they might have different goals and objectives than the government. So, what is the common ground between (them)? If the government looks at the medical tourism numbers and revenue and forgets about the local demand or needs for good and affordable quality healthcare, then this is the issue. So, I think a closer public-private partnership or a collaboration must be on some common agenda for the public good. (Policy researcher for a civil society organisation).

Delivery

There are concerns that medical tourism may attract healthcare professionals to migrate to private hospitals, further reducing the capacity and productivity of the public sector. Despite these concerns about the crowding-out effects of medical tourism on healthcare service delivery, some informants opined that the venture into the industry and subsequent aggressive campaign to attract inbound medical tourists are justified to fill the excess capacity in private hospitals.

Medical tourism currently focuses only on the private sector, and we are not depriving the local people as there is actually excess capacity in the private sector, and we want to utilise the excess capacity for medical tourism purposes. (Chief Executive Officer of a professional body)

I would say that one of the motivations for our private hospitals to include medical tourists in their hospital services is because we need people to utilise our devices and our services. (Academic researcher)

The prime motivation for participating in medical tourism activities is to overcome the underutilisation of private health services following the 1997 Asian financial crisis. Over the years, the prospect of medical tourism's growth has encouraged private hospitals to shift from utilising excess capacity towards expanding capacity to cater to the increasing demand from inbound medical tourists. However, the COVID-19 pandemic and the resulting transboundary travel restrictions have adversely affected the hospitals that had invested to build additional capacity for medical tourism activities, raising questions about the industry's vulnerability.

Let alone the medical tourists, even local patients didn’t go to private hospitals. Since March (2020), I think (the volume of patients) was coming down - some of the private hospitals were operating at 20 per cent capacity, (that is, at) 20 per cent occupancy rate. (Chief Executive Officer of a professional body)

Meanwhile, the informants were divided on the implications of the government's incentivisation of hospitals participating in the industry. Proponents of the medical tourism industry among the informants lauded the incentivisation, capitalising on the healthcare sector's economic prospects. According to them, it would benefit the country through revenue generation and its multiplier effect on the local economy.

But the impact to the economy for each one (Malaysian) Ringgit that a medical tourist spends at a hospital is that they will spend four more (Malaysian) Ringgit in the industry. Your medicine, hotel, transport, meals, shopping, everything. So, if you see they spend one Ringgit, the impact to the economy (is) fourfold. (Top management of MHTC)

They (medical tourists) pay tax; if a medical tourist comes, they will not come alone. They would come with two or three others, right? Two, three of them coming, one of them being a patient and utilise (healthcare services), the rest would spend money on lots of things, in a hotel, eating outside. (Honorary Secretary General of a professional organisation)

On the other hand, an informant objected to the incentivisation for the industry players, as medical tourism is no longer considered a pioneering industry that requires assistance from the government. Meanwhile, another informant stressed that the incentives should be focused on creating an avenue to facilitate investors' participation in the industry rather than providing a tax allowance or tax exemption.

They (private hospitals) need help from MHTC and the government to create an avenue or path for them to enter foreign markets to do their marketing activities. For them to go to the new country as a private hospital is very dangerous and difficult to enter (the market). So, they need more than an MoU between governments; they need the government and MHTC to create the path for them to enter that market. Not much about tax allowance or tax exemption. (Academic researcher)

All informants have differing opinions regarding the type and extent of government incentives to support the industry. As the industry has grown over the years, the incentives should be moulded accordingly, considering the return on investment to the government and other stakeholders.

Despite the differences in opinions, all informants disagreed with any move to expand medical tourism activities to public health hospitals, including the prospect of expanding the role of the private wing in the public hospitals to cater to medical tourists. The involvement of public hospitals in medical tourism may reduce the availability of services for the local population, as these hospitals are already struggling to meet the needs of local patients. Expanding the industry into the public sector could also drain the limited resources of public hospitals.

Not all government hospitals can do it. Some are already busy. We cannot compromise the services to locals and (by implementing such an idea, it may) reduce the capacity of the doctors. Government (hospitals’) priority is to serve locals because (the hospitals are) government funded, right? (Chief Executive Officer of a private hospital chain)

We have to consider so many things, especially in terms of human resources, whether adequate or not, among other things. We may be able to provide the facilities, but is it adequate in terms of human resources? (Senior lecturer from a public university)

Additionally, participating in medical tourism would require public hospitals to implement extra requirements, such as providing translators and enhancing non-clinical aspects, to attract medical tourists.

You are a public hospital with a private wing, but do you have Chinese translators (for medical tourists from China)? Do you have specialists who can speak in Mandarin? Does the food serve suit the (taste of the) patient coming in? These patients are paying money; they’d have different expectations. (Top management of MHTC)

Another implication of trade liberalisation highlighted by several informants is the competition from the commercial presence of for-profit foreign equity participation towards the local private hospitals. Such liberalisation motivates foreign companies to invest in the local healthcare market, thus allowing the expansion of networking among healthcare providers and intensifying the integration of cross-border care. This would consequently encourage the growth of the medical tourism industry. Whilst some welcomed foreign equity into the local market, several other informants raised concerns about the potential negative impacts on local healthcare providers and national interests.

They need funds from overseas. So, that is why the government is opening (the market). If we close the market, there would only be local players, and the market would be small. This is part of capitalism. It is inevitable; you can’t say this is wrong, too. Because if you don’t allow foreigners to invest in Malaysia for certain sectors, then how can you grow? (Honorary Secretary General of a professional organisation)

I think healthcare is a public utility, national service, and essential service and putting the private hospital under 100 per cent foreign equity ownership may erode our sovereignty and our effort to protect our national security. (Senior fellow in an academic institution)

It’s not a good idea because it weakens our local private hospitals. But (with regards to) our local private hospitals, although I’m against them, at least their profits circulate in the country, (still) better than this. (Member of a civil society organisation)

Financing

Several informants acknowledged the potential negative impact of medical tourism on the dichotomous healthcare system. They argued that promoting medical tourism would exacerbate the polarisation of access within a two-tier system where private hospitals are mainly accessible to patients from higher-income groups whilst public hospitals are overcrowded with those from lower socio-economic backgrounds.

There would be an impact to healthcare access to Malaysians, how it would impact the poor, also issues of national unity when the two-tier healthcare system, one that caters the rich and foreign, one that caters the poor and local. (Senior fellow in an academic institution)

MOH always emphasise that public healthcare is for helping the poor. It’s true because they can’t afford private healthcare. (Policy researcher for a civil society organisation)

They also highlighted the differential pricing of services provided to medical tourists, which may be higher than for local patients.

Yes, we have different pricing; locals usually enjoy a much lower price for certain services. For foreigners, if they can pay, they should be charged more. For example, we charge Chinese (medical tourists from China) patients full price. In contrast, we usually have a special local package for local patients, for example. (Chief Executive Officer of a private hospital)

The cost for the locals would be lower compared to the foreign patients. It will improve access of our local people to this private sector. (Senior health system researcher in a government research centre)

And if there is going to be differential pricing, there should be differential pricing in the luxury services offered rather than the actual clinical services. You can differentiate if you put the person into a one-bedded room versus a four-bedded room or the quality of food you offer. However, you should not have differential pricing based on nationality. Everybody should have the universal human right to basic healthcare. (Senior fellow in an academic institution)

Conversely, an informant argued that medical tourism offers additional patients to private hospitals, thus allowing them to reduce service charges through economies of scale.

It (medical tourism) could allow private hospitals participating in the industry to reduce the cost of services provided to the locals through economies of scale. Medical tourism provides additional volume to the private hospital, which could further lower the charges. (Chief Executive Officer of a private hospital)

Informants largely agreed that out-of-pocket payments are the primary source of healthcare financing in the medical tourism industry, apart from international insurance funds. Some informants highlighted the interrelationship between transboundary health insurance portability and hospital accreditation by the international accreditation body.

They (third-party payers such as government, employers, and insurance companies) don't simply allow the patients to seek treatment at facilities with questionable quality. For example, some insurance companies would allow transfer of coverage from their country of origin to the destination countries - given that the hospital providing care must be accredited by an international accreditation body. This is why you can see most hospitals participating in medical tourism in Malaysia chose an international accreditation body over the local (MSQH) - some even seek accreditation from both - even though the local body is actually at par with the international body. (Senior lecturer from a public university)

Accreditation may not necessarily equals to quality, but it could serve as a 'proxy' to the hospital's commitment to ensure the quality of service. Insurance companies, for example, would not simply allow medical tourists to seek care at any hospital. They would look at the hospital's credentials, including accreditation by the international body. (Medical Director of a private hospital)

In addition to being a plus point in terms of marketing where accreditation by international body would attract medical tourists, it (international body's accreditation) is also being preferred or required by some insurance companies for the coverage of benefits. (Chief Executive Officer of a private hospital)

Regulation

Most informants cited international hospital accreditation as having positive implications for the industry and healthcare system. Accreditation from accreditation bodies improves quality and assurance for potential inbound medical tourists. Due to the requirements set by these bodies, accreditation by international accreditation bodies contributes to better service quality. Additionally, it can help promote accredited hospitals, as accreditation is included as one of the criteria of selection by third-party payers such as insurance companies, governmental payers, or employers.

Accreditation is one of the requirements for MHTC membership. For us, at least we know, we can say that you comply with the minimum standard. If the accreditation body - MSQH (Malaysian Society for Quality in Health) (for example) says enough, we consider it enough. (At least) minimum. (Top management of MHTC)

I do not deny (that) meeting the accreditation requirement does in a way positively impact the hospital service delivery because of the requirement. They also improve their medical and non-medical services when they comply with the requirements. (Academic researcher)

Another aspect that informants from the private health sector raised was the strict regulations imposed by the government. Whilst it is acknowledged that the Ministry of Health plays a vital role as the regulator, some informants expressed concern that the private health sector is currently being highly regulated to the extent that it raises the cost of operation for private hospitals.

Over the years, the regulations for private hospitals have become so tight that the cost of setting up a hospital has increased significantly. One of the regulations by MOH is that you can only offer a service if you have the facility and the resident doctors. So, that means that everybody is investing in the same resources, and there’s an overinvestment of resources in healthcare, which the DG (Director-General of Health) like to talk about - too many PET (positron emitted tomography) scans in private hospitals, et cetera. But that is because they have also regulated that everybody must have their own. (Chief Executive Officer of a private hospital)

Private healthcare is regulated by more than 51 laws and regulations. So, it is very, very micromanaged, and highly regulated. (Honorary Secretary General of a professional organisation)

Nonetheless, all informants agreed that regulations and hospital accreditation enhance the care provided by private healthcare providers, including hospitals participating in medical tourism.

Human capital

Several informants acknowledged the impact of medical tourism, which led to the movement of healthcare professionals from the public health sector to the private health sector. Some informants mentioned the term ‘brain drain’ whilst discussing this scenario.

The first noticeable impact is the brain drain, which attracts mostly specialists going from a public hospital to a private hospital, simply because there is a demand for their services. (Senior fellow in an academic institution)

Our government health services are having a problem of brain drain. Around 75 per cent are in private, only about 25 per cent are in government (sector). (Member of a civil society organisation).

Furthermore, an informant opined that migration to the private sector would involve certain specialties that could generate better income and are in demand by inbound medical tourists. The demand may not be in line with the health demands and needs of the local population.

The type of specialisation that can’t make money in the private sector would remain mainly in the public sector and those surgical-based specialities more popular among the private sectors. (Senior health system researcher in a government research centre)

Additionally, the migration of experienced specialists to the private health sector could affect the training of future doctors and specialists, which is mainly conducted in public health institutions. Although some private health institutions have started their training programmes, the volume of patients in private hospitals may be smaller compared to public hospitals, which may impact the training of future specialists in terms of their adequacy of experience in managing patients.

There is still a little bit of worry whether the private sector will be able to train doctors and healthcare providers, whereas traditionally, it has always been done by the government institutions. But, nevertheless, I think over a period of time, this will catch up. (Medical Director of a private hospital)

However, proponents of medical tourism among the informants argued that medical tourism may contribute to the retention of specialists in local private hospitals. This would also benefit locals, who still form the majority of patients utilising the services provided by these hospitals.

It boils down to the structure of our private hospital because our hospitals are serving local patients, that’s the bread and butter of their business, but along the way, like along the way kind of business we are serving medical tourists. (Academic researcher)

Equitable access

In a departure from Pocock and Phua’s policy implications framework, a specific theme for the issues of access to healthcare from the perspective of local consumers has emerged from the thematic analysis in this study. Most informants have prominently highlighted this implication, sometimes overlapping with other implications reported earlier. The informants who opposed the promotion of the industry argued that medical tourism may exacerbate the underlying maldistribution of secondary and tertiary health services.

So, of course, you will have a lot of concentration of healthcare providers in the city because medical tourism is largely at urban centres with attraction sites and proper transportation. (Medical Director of a private hospital)

Three informants from health-related civil society organisations expressed their opposition to the industry, citing that it will result in the prioritisation of services and the rationing of the healthcare system according to economic and financial importance. One of these informants had expressed a strong opinion concerning this matter, further stressing the incompatibility between healthcare delivery and the profit-driven nature of medical tourism.

The impact is detrimental due to the drawing away of medical resources from the provision of healthcare in the public sector, and medical resources being rationed according to economic and financial criteria rather than medical criteria. (Member of a civil society organisation)

The health sector, in general, is about taking care of people for the people’s benefit, whereas when we talk about tourism, the words themselves suggest something for profit making. Health and profit usually don’t go together. (Senior health system researcher)

Establishing medical tourism-centred hospitals that primarily treat inbound medical tourists could raise health equity concerns as it would exacerbate the polarisation of the two-tier healthcare system in a destination country like Malaysia. However, other informants deem this scenario unlikely, given that most participating hospitals in Malaysia still cater to the demand of local patients, who form most of their clients.

Impact of COVID-19 on medical tourism and the healthcare system

The pandemic has had a devastating impact on both the medical tourism industry and the healthcare system. Similar to other industries reliant on cross-border activities such as international trade and tourism, interventions such as travel restrictions and lockdowns have reduced the volume and revenue from medical tourism activities. The informants stressed that the pandemic has exposed the vulnerability of the medical tourism industry to external factors that could affect cross-border travel and the economy. Therefore, there should be a change in strategy towards the industry to strengthen its resilience and ensure the appropriate return of investment from the industry to the country. As one of the informants suggested, this may be done by integrating medical tourism activities into a broader scope of services, such as elderly care.

Despite the setbacks due to COVID-19, some informants saw the positive side in which this pandemic may accelerate closer public-private collaboration. This pandemic has also shown the importance of a closer public-private partnership in healthcare to manage the available resources efficiently. It has also accelerated the partnership and may pave the way for better collaboration towards achieving the goal of equitable access to healthcare services.

Due to COVID-19, we (the public sector) have to decant some of our services. Maybe we can divert some of these patients under public-private partnership programme. “We cannot manage these patients; we go to your hospital (private), and you give us a special rate, but you are guaranteed so-called customers”. So (there will be) guaranteed volume, but you must lower your prices. I mean, that kind of arrangement (certainly) can be done. (Government servant of a division under Ministry of Health)

I foresee some issues, for example, the COVID-19 vaccination programmes. It’s going to be very difficult if only the public sector gives out the vaccines with adequate coverage. So, they will need to get the private health sector involved.... (Chief Executive Officer of a private hospital)

## Discussion

After more than a decade of extensive promotion of medical tourism, we revisited the industry's policy implications within Pocock and Phua’s original framework. In the context of Malaysia’s current healthcare system and the COVID-19 pandemic, we explored the multifaceted interrelationship between medical tourism and the national healthcare system. We have identified several factors affecting medical tourism and the national healthcare system through in-depth interviews with informants from various backgrounds. These factors may directly impact medical tourism activities or indirectly through their impact on the healthcare system, which in turn would affect the industry and its sustainability. These factors range from aspects of governance through the health sector commitments in trade agreements and collaboration between the public and private health sectors, government, healthcare service delivery, health financing, and regulations of the industry and the health sector in general.

The Malaysian government’s commitments to health-related trade agreements impact medical tourism and the national healthcare system. For instance, Mode 1 (cross-border supply) under GATS allows hospitals involved in medical tourism to conduct remote consultations through telemedicine with their patients for pre-treatment consultation or follow-up after performing certain procedures [26]. Telemedicine has become more prevalent in medical tourism, especially after the COVID-19 pandemic and the cross-border travel restrictions implemented by the government. The existing Telemedicine Act 1997 regulates the practice of telemedicine in Malaysia, but it has not been revised to current standards. Nonetheless, the Malaysian Medical Council has formulated a virtual consultation advisory in response to the need for telemedicine in the COVID-19 situation. The advisory has been rescinded and replaced by the Malaysian Medical Council Guideline on Telemedicine in 2024 [27].

Mode 1 also covers transboundary health insurance policies, which could benefit medical tourism activities. However, this also depends on the portability of health insurance [12] and the nature of certain existing health insurance plans that explicitly or implicitly discriminate against treatment abroad [28]. Nevertheless, the issue of health insurance portability could be overcome by encouraging private international insurance companies to venture into the domestic economy to cover the medical insurance of medical tourists.

Meanwhile, Mode 3 (commercial presence) could also boost the medical tourism industry by encouraging foreign companies to invest in Malaysia and setting up private hospitals with up to 100 per cent equity ownership. Singapore, as one of the historically leading destination countries, attracted foreign investment in internationally renowned flagship hospitals such as John Hopkin International Medical Centre in its effort to promote medical tourism [29]. However, increased foreign direct investment (FDI) will bring both opportunities and challenges, along with risks and benefits, to both developed and developing countries and those exporting and importing investment. Unlike portfolio investment, FDI is undertaken to exert control over the enterprise being invested in. These binding trade agreements and control by foreign equity could further reduce the government’s policymaking space to act for the benefit and rights of its people. Therefore, the terms and commitments in these agreements must be carefully negotiated, with people’s interests being prioritised over the potential lucrative economic drive.

Stakeholder collaboration in the medical tourism industry in Malaysia has also encouraged the industry's growth through the pivotal role played by MHTC as the facilitator for marketing strategies abroad and bridging the private sectors with various relevant government agencies. Several other destination countries have developed similar national booster public-private partnerships, such as the Korea International Medical Association, the Taiwan Taskforce for Medical Travel, and the Philippine Medical Tourism Program [29]. Collaboration between various stakeholders from the public and private sectors is not limited to the medical tourism industry. Broader collaboration in public-private partnerships is also crucial towards ensuring that Malaysia can achieve and maintain universal healthcare in this country through healthcare reform, especially in terms of financing mechanisms.

While integrated public-private health service delivery through a proper health financing mechanism could improve the efficiency of healthcare service utilisation, policymakers should be cautious so as not to increase the pressure on the already relatively small government expenditure on healthcare through public financing of private delivery to achieve universal health coverage [30]. Apart from financing, another aspect that should be scrutinised is whether public-private partnerships could overcome regional disparities since most private health providers are concentrated in urban regions. If new facilities are needed to overcome the maldistribution of healthcare facilities, questions arise on how the cost of setting up such new facilities would be managed, whether it would be subsidized by the government or shared by the private practitioners interested in enrolling in the partnership programme.

Public-private partnerships in the health sector can be further strengthened by formulating a proper financing mechanism that could allow efficient utilisation of all available resources from both sectors. One of the approaches is to embark on health financing reform based on strategic purchasing [31]. Strategic purchasing involves negotiations between the government and private health providers to fund the services available at appropriate costs. This partnership can also increase coordination between both sectors, thus preventing duplication or redundancy, ensuring continuity of care, and avoiding underutilisation of high-end technology. This can also increase the efficiency of the provision of care, enhance equity in the distribution of resources, promote the quality of the delivery of care, and manage expenditure growth between the public and private sectors. It also improves the accountability and transparency of purchasers and providers to the population. Creating an independent body for cost management and cost containment in the public and private health sectors could further enhance such public-private partnerships. Such initiatives have been long debated and scrutinised among policymakers and other stakeholders in healthcare. However, it has been delayed for a long time due to multiple financial, regulatory, structural, and political challenges that the government faced.

On a different note, the government’s multiple roles in healthcare as the funder and provider of healthcare in the public health sector, as the regulator of the healthcare system, and as a significant investor in the for-profit private health sector may lead to conflicting interests that may be detrimental to Malaysian citizens’ access to equitable healthcare. Furthermore, this could further exacerbate the dichotomous nature of national healthcare as the government, through its government-linked investment company, would invest more in the growth of private hospital chains whilst having limited resources to cater to the healthcare needs in the public health sector. The dichotomy would persist unless there is a proper form of cross-subsidisation from the revenue generated by these private hospitals to improve the condition in public hospitals or having the tax revenue from these private hospital chains be identified and explicitly allocated for healthcare.

Regulation of hospitals participating in medical tourism and the requirement for accreditation for private hospitals planning to participate in medical tourism directly impact the industry and may also indirectly affect the whole healthcare system. Regulatory enforcement and accreditation may increase patients’ safety and standard of care. These regulations and accreditation requirements also protect medical tourists from fraud and illegal or substandard procedures.

Several regulatory levers could be implemented at various levels, either by the departure countries or destination countries, as well as multilateral regulations. Meanwhile, the countries that are signatories to GATS are bound by the commitments that they have signed and ratified. Regulations by the departure countries ranged from (i) direct control of the type of medical care covered under the government-prompted medical tourism, (ii) preventing or limiting the private insurer-prompted medical tourism, (iii) interventions through the provision of information on the choice of treatment or that directly restricts them, and (iv) regulating the intermediaries or ‘facilitators’ (such as MedTreat in the United States of America or Globe Health Tours in the United Kingdom) or the private sector through guidelines and accreditations. Meanwhile, destination countries could also regulate the industry through various legislation to protect their national interests and ensure that local patients are not deprived of their healthcare needs [32]. However, stringent regulations may result in unnecessary regulatory burdens for the participating hospitals and could hinder them from sustaining their participation in the industry. In terms of public-private partnerships in medical tourism activities, the now-defunct multi-agency partnership Singapore Medicine model has been followed by many countries to promote medical tourism in their countries [29]. The partnership was launched in 2003 to enhance the island state’s standing in the international arena and as part of the push to promote Singapore as a regional hub for medical excellence [33].

Medical tourism also has a favourable implication by enhancing the standard of care through the retention of highly skilled health professionals, the requirement for accreditation, and the drive to gain a competitive edge in attracting medical tourists, which will also benefit local patients who utilise private healthcare services [34]. Participation in the medical tourism industry would also increase the pool of patients treated in private hospitals. It would, therefore, allow these private hospitals to invest more in cutting-edge treatment modalities with higher returns on investment due to economies of scale. The benefit could also be directly extended to local patients through cross-subsidisation of the local patients seeking treatment at private hospitals.

Several human capital issues in healthcare may also impact the medical tourism industry and the healthcare system. One of the most prominent issues is the apparent lack of coordination in management and human resource planning between ministries and the private health sector for the supply of future human capital. This could lead to a mismatch between the supply and demand of human capital in healthcare. Eventually, this culminated in a relative oversupply of medical graduates in this country over the last decade, with limited places for their internship or horsemanship training. In Malaysia, all medical graduates are required to undergo a two-year period of resident medical practice, as stipulated by the Medical Act 1971. Fresh medical graduates are required to complete their housemanship under the supervision and guidance of clinical specialists in public hospitals. Whilst there is a relative oversupply of medical graduates, there are reportedly inadequate specialists to serve the public and train the fresh medical graduates during their housemanship.

Currently, housemanship training in Malaysia is only provided in public hospitals under the Ministry of Health and teaching hospitals under the Ministry of Higher Education. Therefore, this matter could be worsened by the ‘poaching’ of specialists trained in public education institutions from the public to the private health sector. Consequently, there would be a higher houseman-to-specialist ratio, which may affect the training of the housemen. The medical tourism industry might lead to the inequitable use of public resources, either in the direct utilisation of public facilities for medical tourism activities or indirectly from the ‘poaching’ of skilled health professionals trained in the public education system. Whilst medical tourism activities in Malaysia are driven by private hospitals, the ‘poaching’ of skilled health professionals from the public education system persists. Similar impacts are also identified in Thailand and India, two prominent destination countries in medical tourism [15,35,36].

Another potential negative implication is that this industry could be the source of inequity in access to healthcare due to the problems of ‘brain drain’ from the public sector to the private sector and consequently increasing rural deprivation. Higher pay and advanced medical technologies in the hospitals participating in medical tourism are pulling factors to lure health professionals from the public sector [12,37,38]. Furthermore, the internal migration of specialists, in particular, could further deprive rural areas of receiving quality care, as the facilities participating in medical tourism are primarily located in urban centres [39-41]. The negative implications of ‘brain drain’ as a result of migration from the public to the private health sectors could be worsened if the issue of a dichotomous, two-tier healthcare system is not properly addressed. Therefore, measures must be taken to address regional disparities in access to healthcare services should the government decide to actively promote the industry's growth.

The inequity was further exacerbated by the COVID-19 pandemic, in which the burden of care was shifted towards prioritising the management of COVID-19 patients at public hospitals during the height of the pandemic, as the private hospitals are largely not properly equipped to manage infectious diseases of such proportion. Furthermore, the pandemic has affected livelihoods at the individual level and the economy in general. Without proper health financing mechanisms and a high proportion of OOP payments among the population, there could be a shift towards the utilisation of government-funded hospitals. This may worsen the overburdened and underfunded public health sector.

Addressing the underlying issues of equity in access to healthcare services within the two-tier system would overcome the potential negative implications of the growing medical tourism industry, which could have worsened the situation. In Malaysia, the healthcare privatisation policy had taken place more than a decade before the medical tourism industry came onto the agenda [42]. Therefore, it has been argued that the medical tourism industry should not be blamed for deficits in developing countries' healthcare systems, as they existed before this development and were primarily due to internal factors [43,44]. Although it has also been argued that medical tourism may exacerbate the inequalities in the healthcare system in destination countries and could potentially undermine the public health sector, it remains essential to contextualise its impact [14]. On the other hand, healthcare reform is direly needed to address the growing concerns about equity, efficiency, rising healthcare costs, and financial constraints and to balance divergent and conflicting policy principles.

Similar to recreational tourism, medical tourism was also adversely affected by the COVID-19 pandemic and the resulting travel restrictions. Therefore, there is a need to develop a resilient medical tourism industry that would also benefit the local population. Incorporating medical tourism activities into a broader framework, such as promoting elderly care within the retirement destination package for expatriates, could ensure its sustainability instead of relying solely on medical tourism activities. Expanding medical tourism into the existing Malaysia My Second Home programme could ensure the industry's resilience in anticipation of another pandemic that would affect cross-border travel resulting from travel restrictions imposed as one of the measures to control and mitigate the impact of such an event. The programme is promoted by the Malaysian government through the Immigration Department of Malaysia, allowing non-Malaysians to retire and live in Malaysia for an extended period of time.

Recalibrating the industry's approach and structural reform of the healthcare system are the ways forward to establish a resilient medical tourism industry within a sustainable and equitable healthcare system. Capacity building and diversification of services offered by industry players should be aligned with the Ministry of Health Malaysia's strategies to cater to the local population's health needs. As Malaysia is heading towards an aged nation status, there should be a focus on establishing streamlined rehabilitation and geriatric care whilst strengthening the existing niches offered in medical tourism. The facilities and treatment modalities in these specialties should also benefit local consumers, as these private hospitals participating in medical tourism also treat the locals. Furthermore, aligning the ‘supply’ of medical tourism with the local populations' health needs would ensure specialists' training is prioritised proportionately.

More importantly, structural reform, including restructuring the health financing mechanism and optimising health service delivery through a stronger public-private partnership, is essential to ensuring an equitable and sustainable healthcare system. The Malaysian government has expressed its commitment towards achieving this goal through the Health White Paper [45], which was tabled and passed by the Malaysian Parliament on June 15, 2023. Although this document does not outline its position in the medical tourism industry, we anticipate that implementing all pillars under its strategic framework may overcome the potential negative implications of the industry towards the healthcare system and help build a more resilient and equitable healthcare system.

For instance, under Pillar 1, the government aspires to develop a comprehensive framework for public-private partnerships in secondary and tertiary service provision, including mechanisms to ensure value-based services with balanced coverage of specialists across facilities and geographies. The document also highlighted the proposal to develop an efficient procurement framework by establishing value-based payment models, demand-supply mapping, strengthening the strategic purchaser function, and other key components.

Consequently, this may contribute to allocative efficiency by reducing the workload at public hospitals and efficiently increasing the utilisation of private health facilities. Therefore, this may reduce the interest of local private hospitals in participating in medical tourism to cater to excess capacity. Instead, the hospitals that have already been established for medical tourism activities may resume or expand their capacity to cater to medical tourists' health demands. It is also possible that the government may introduce taxes on medical tourists to generate income, which would be redistributed to finance the national healthcare system.

Effective public-private collaboration would ensure that healthcare services are delivered at a reasonable cost across every level of care and help to balance resource utilisation across the public, private, and non-profit sectors. The Health White Paper also intends to review and update the accreditation and performance standards for the public, private, and non-profit sectors. This would further enhance the credibility of the health sector in Malaysia while ensuring the standard of care.

Strengths and limitations

The main strength of this study is the comprehensiveness of the scope covered to achieve its objective. Building on Pocock and Phua's published framework, we explored the existing domains and beyond extensively. The emergent theme of ‘equitable access’ highlights the concerns of various stakeholders towards the underlying issue of equity in health, which was worsened by the COVID-19 pandemic and the resulting socio-economic impacts from the pandemic. Although the basic design does not offer explanatory capabilities or identify the relationships between the pre-existing factors that facilitate the growth of the industry and the impacts of the practice, the original framework does provide a valuable scheme to identify and compile additional policy-relevant insights. Furthermore, our study is conducted within the Malaysian context, which was also included as one of the countries studied in the original study from which the conceptual framework was derived. To our knowledge, this study is the first conducted on the policy-relevant implications of medical tourism in the aftermath of the COVID-19 pandemic. This allows us to gain insights into how the pandemic impacts the dynamics of the interrelationship between the industry and the national healthcare system from the perspective of the relevant stakeholders.

Due to the nature of the qualitative case study design, the findings from this study may not be generalisable to other destination countries with different national healthcare systems and health financing mechanisms. Nonetheless, qualitative research seeks to achieve transferability instead of generalizability. Furthermore, the insights from the current study and the original Pocock and Phua's framework are intended to promote future research rather than being prescriptive in nature.

The broad scope of topics covered in the interviews, encompassing the multifaceted relationship between medical tourism and the healthcare system, resulted in more interview sessions involving representatives from various stakeholders to achieve the level of saturation.

## Conclusions

The current analysis demonstrates that the growth of medical tourism is welcomed by most stakeholders with caution due to its vulnerability to external factors such as the COVID-19 pandemic and the negative impact on equitable access to the healthcare system if not properly regulated. Meanwhile, the industry players expressed concern regarding strict regulations imposed by the government, which may escalate the cost of operations for private hospitals. On the other hand, three out of four informants representing civil society organisations are against the industry, reflecting their stand against health commodification from the social justice point of view.

A robust medical tourism industry within a sustainable and resilient healthcare system would benefit medical tourists and the local population in the destination countries. This industry could also enhance the quality and standard of care by encouraging healthy competition among its participants. Eventually, this would benefit the economies of the destination countries in general.

Concurrently, policy implications arising from the industry remain relevant and should be addressed through a comprehensive structural reform of the national healthcare system involving stakeholders from the public and private health sectors. The proposed Health White Paper by the Malaysian government demonstrates a determined political will towards comprehensive structural reform of the healthcare system. This initiative would address underlying health inequities and foster effective public-private collaboration, propelling the nation towards universal healthcare.

## Appendices

Topic guide

Introduction

Thank you for participating in this study. I am a Doctor of Public Health (DrPH) candidate from the Department of Social and Preventive Medicine, Faculty of Medicine, University of Malaya. I am also an employee of the Ministry of Health, Malaysia, who is currently on study leave under a government scholarship (Hadiah Latihan Persekutuan).

I am currently doing my thesis on the medical tourism industry, focusing on its implications for the healthcare system in this country. This interview session is part of the study to explore the implications of medical tourism for the Malaysian healthcare system from the perspective of relevant stakeholders involved in the medical tourism industry in Malaysia. This also includes the impacts of the COVID-19 pandemic on both and the way forward in the post-pandemic future.

In addition, this interview will help explore stakeholders' opinions in the Malaysian medical tourism industry regarding the potential benefits and adverse effects of medical tourism on the Malaysian healthcare system.

The participant’s information sheet provided briefly explains how to conduct this interview. Please take your time to carefully read it; you may ask further questions for clarification. This interview can be conducted either in Bahasa Malaysia or English, depending on your preference.

Each session will take approximately 45 minutes to 1 hour to complete. All participants are guaranteed their anonymity and confidentiality.

Part 1

The first part of this interview will cover your professional background and your personal view on the prospect of medical tourism in this country.

1.     For the record, can you please provide a brief introduction of your professional background in terms of qualifications, expertise, years of service, and experience in medical tourism?

2.     What is your opinion regarding medical tourism in Malaysia?

Part 2

The second part of this interview will cover the multifaceted relationship between medical tourism and the national healthcare system, exploring these two broad aspects:

1.     How do the institutional characteristics of the national healthcare system shape the demand for medical tourism in Malaysia and other destination countries?

2.     How do these characteristics shape the very nature of the impact of medical tourism on the healthcare system?

Participants will be asked open-ended questions in semi-structured key informant interviews on their perceptions of the implications of medical tourism based on these domains:

1.     Governance in the trade and health sectors

a.     Expansion of ministerial responsibilities and convergence of inter-ministerial relationships

b.     Number and content of health sector commitments in multi- and bilateral trade agreements

c.     Regional trade blocs promoting trade in health services

d.     Creation of medical tourism travel visas

Question: What is your opinion regarding the government’s involvement in boosting the medical tourism industry in Malaysia in the aforementioned aspects of governance in the trade and health sectors?

2.     Delivery in the private versus public sector

a.     Utilising of existing private sector supply

b.     Growth of the private health sector and increase in for-profit healthcare delivery

c.     Government subsidies for private sector growth through tax breaks and other incentives

d.     Foreign direct investment in health infrastructure (creation of ‘offshore’ medical facilities with deep ties to neighbouring health systems and foreign investment)

Question: How does the medical tourism industry affect healthcare delivery in both public and private healthcare services?

3.     Healthcare financing and consumerism

a.     Out-of-pocket payments

b.     Interest in internationally portable health insurance

c.     Growth of consumer-driven healthcare, competition for patients based on price

d.     Differential pricing for foreign patients and local consumers

Question: What is your comment on the implications of the medical tourism industry for healthcare financing and consumerism in healthcare?

4.     Human resources and specialist training

a.     International emigration of health workers, particularly specialists

b.     Intra-country migration from rural to urban areas

c.     Distribution of specialists between the public and private health sectors

d.     Future human resource capacity (re: training, availability, professional-to-population ratio)

Question: What do you think of the emerging medical tourism industry's implications for the dynamics of human resources and the field of specialties?

5.     Regulation of quality control

a.     Public and private sector quality control: different quality accreditation channels at the national and international levels

b.     International accreditation of health facilities (e.g. Joint Commission International)

c.     Number of medical tourist visits facilitated by brokers

Question: What is your opinion about the implications of the medical tourism industry for affecting the quality of services provided by the private and public health sectors?

Part  3

The third part covers the impact of the COVID-19 pandemic on the dynamics of the relationship between medical tourism and the healthcare system.

1.     How does the COVID-19 pandemic impact the multifaceted relationship between medical tourism and the Malaysian healthcare system?

2.     What is the best way forward for the future of the medical tourism industry post-COVID-19?

Part 4

The final part (fourth) covers the concluding remarks and recommendations by the informant.

1.     How will medical tourism affect the national healthcare system and the local population’s access to healthcare services?

2.     How can you or your organisation, in tandem with other stakeholders, contribute to a better healthcare system and the growth of medical tourism in this country?

3.     Is there anything else you want to add before we end this session?

4.     Would you suggest other informants that you find suitable for this interview?

## References

[1] Lunt N, Smith RD, Exworthy M, Green ST, Horsfall DG, Mannion R (2011). Medical tourism: Treatments, markets and health system implications: a scoping review. https://pure.york.ac.uk/portal/en/publications/medical-tourism-treatments-markets-and-health-system-implications.

[2] Cortez N (2008). Patients without borders: The emerging global market for patients and the evolution of modern health care. Ind L J.

[3] Carrera PM, Bridges JF (2006). Globalization and healthcare: understanding health and medical tourism. Expert Rev Pharmacoecon Outcomes Res.

[4] Edelheit J (2008). Defining medical tourism or not?. Medical Tourism Magazine.

[5] Lunt N, Carrera P (2010). Medical tourism: assessing the evidence on treatment abroad. Maturitas.

[6] Jagyasi P (2008). Defining medical tourism - another approach. Medical Tourism Magazine.

[7] Hopkins L, Labonté R, Runnels V, Packer C (2010). Medical tourism today: what is the state of existing knowledge?. J Public Health Policy.

[8] Connell J (2015). Chapter 2: medical tourism - concepts and definitions. Handbook on medical tourism and patient mobility.

[9] Ormond M, Mun WK, Khoon CC (2014). Medical tourism in Malaysia: how can we better identify and manage its advantages and disadvantages?. Glob Health Action.

[10] Wong Lai Lin, Mary Mary (2008). The development of health care system in Malaysia - with special reference to government health services, 1970-2000. http://scholarbank.nus.edu.sg/handle/10635/13250.

[11] Ministry of Health Malaysia (2024). MOH. Medical Advertisement Board Advertising Guideline for Healthcare Facilities and Services (Private Hospitals, Clinics and Medical Laboratories). In: Board MA, editor. Kuala Lumpur. Advertising guideline for healthcare facilities and services (private hospitals, clinics and medical laboratories).

[12] Chee HL (2007). Medical tourism in Malaysia: international movement of healthcare consumers and the commodification of healthcare. Asia Research Institute Working Paper No. 83.

[13] Dahlui M, Aziz NA (2012). Developing health service hub in ASEAN and Asia, region country report on healthcare service industry in Malaysia. Developing ASEAN Economic Community (AEC) into a global services hub.

[14] Ormond ME (2013). Neoliberal governance and international medical travel in Malaysia.

[15] NaRanong A, NaRanong V (2011). The effects of medical tourism: Thailand's experience. Bull World Health Organ.

[16] Safurah J, Kamaliah M, Khairiyah A (2013). Malaysia health system review. https://iris.who.int/bitstream/handle/10665/206911/9789290615842_eng.pdf.

[17] Yu CP, Whynes DK, Sach TH (2008). Equity in health care financing: the case of Malaysia. Int J Equity Health.

[18] Bakar NS, Manual A, Hamid JA (2019). Socioeconomic status affecting inequity of healthcare utilisation in Malaysia. Malays J Med Sci.

[19] (2024). Healthcare Travellers Statistics. MHTC. Healthcare Travellers Statistics: Malaysia Healthcare Travellers Council.

[20] Malaysia Healthcare Travel Industry (2021). Malaysia Healthcare Travel Industry blueprint 2021-2025.

[21] Ministry of Health Malaysia (2015). National Health and Morbidity Survey. National health and morbidity survey 2015.

[22] Rechel B, Doyle Y, Grundy E, McKee M (2009). How can health systems respond to population ageing?. https://iris.who.int/handle/10665/107941.

[23] Ormond M (2011). Shifting subjects of health-care: placing "medical tourism" in the context of Malaysian domestic health-care reform. Asia Pac Viewp.

[24] Guest G, Bunce A, Johnson L (2006). How many interviews are enough? An experiment with data saturation and variability. Field Methods.

[25] Pocock NS, Phua KH (2011). Medical tourism and policy implications for health systems: a conceptual framework from a comparative study of Thailand, Singapore and Malaysia. Global Health.

[26] Alexander AA, Thawornchaisit P (2019). Global trading in health services: potential trade and system-based challenges for traders. J Thai Interdiscip Res.

[27] Malaysian Medical Council (2024). Guideline on Telemedicine. Malaysian Medical Council guideline on telemedicine.

[28] Mattoo A, Rathindran R (2006). How health insurance inhibits trade in health care. Health Aff (Millwood).

[29] Ormond ME, Mainil T (2015). Chapter 15: government and governance strategies in medical tourism. Handbook on medical tourism and patient mobility.

[30] Kumar R (2019). Public-private partnerships for universal health coverage? The future of "free health" in Sri Lanka. Global Health.

[31] Barcellona C, Khor SK, Lim J (2023). Challenges and opportunities for regional collaboration for strategic purchasing in Southeast Asia. Lancet Reg Health Southeast Asia.

[32] Cohen IG (2012). How to regulate medical tourism (and why it matters for bioethics). Dev World Bioeth.

[33] Lim MK (2005). Transforming Singapore health care: public-private partnership. Ann Acad Med Singap.

[34] Yusof N, Rosnan H (2020). Serving the medical tourists in Malaysia: are local patients being put the second?. Asian J Environ-Behav Stud.

[35] Hazarika I (2010). Medical tourism: its potential impact on the health workforce and health systems in India. Health Policy Plan.

[36] Ramírez de Arellano AB (2007). Patients without borders: the emergence of medical tourism. Int J Health Serv.

[37] Chinai R, Goswami R (2007). Medical visas mark growth of Indian medical tourism. Bull World Health Organ.

[38] Arunanondchai J, Fink C (2006). Trade in health services in the ASEAN region. Health Promot Int.

[39] Connell J (2006). Medical tourism: Sea, sun, sand and … surgery. Tour Manag.

[40] Wibulpolprasert S, Pachanee CA, Pitayarangsarit S, Hempisut P (2004). International service trade and its implications for human resources for health: a case study of Thailand. Hum Resour Health.

[41] Blouin C (2007). Trade policy and health: from conflicting interests to policy coherence. Bull World Health Organ.

[42] Wilson A (2010). Medical tourism in Thailand. Asian biotech: ethics and communities of fate.

[43] Pennings G (2006). Ethics without boundaries: medical tourism. Principles of health care ethics.

[44] Chee HL (2010). Medical tourism and the state in Malaysia and Singapore. Glob Soc Policy.

[45] Ministry of Health Malaysia (2023). MOH. Health White Paper For Malaysia. Health white paper for Malaysia.

